# Genetic characterization of HIV-1 viruses among cases with antiretroviral therapy failure in Suzhou City, China

**DOI:** 10.1186/s12981-023-00540-0

**Published:** 2023-06-28

**Authors:** Zefeng Dong, Zhihui Xu, Ying Zhou, Runfang Tian, Kai Zhou, Di Wang, Xuerong Ya, Qiang Shen

**Affiliations:** 1 Suzhou Center for Disease Control and Prevention, Suzhou, 215004 China; 2 Jiangsu Provincial Center for Disease Control and Prevention, Nanjing, 210003 China

**Keywords:** HIV, Drug resistance, ART, *Pol* gene, Recombinant

## Abstract

**Background:**

This retrospective study aimed to characterize the distribution of HIV-1 genotypes and the prevalence of drug resistance mutations in people with antiretroviral treatment (ART) failure in Suzhou City, China.

**Methods:**

Pol gene of HIV-1 viruses in blood samples of EDTA anticoagulants from 398 patients with failed antiviral treatment was successfully amplified by using an in-house assay. Drug resistance mutations were analyzed by using the Stanford HIV Drug Resistance Database system (https://hivdb.stanford.edu/hivdb/by-mutations/). HIV-1 genotypes were determined by the REGA HIV subtyping tool (version 3.46, https://www.genomedetective.com/app/typingtool/hiv). Near full-length genomes (NFLG) of HIV-1 viruses were obtained by next generation sequencing method.

**Results:**

Sequences analysis of the *pol* gene revealed that CRF 01_AE (57.29%, 228/398) was the dominant subtype circulating in Suzhou City, followed by CRF 07_BC (17.34%, 69/398), subtype B (7.54%, 30/398), CRF 08_BC (6.53%, 26/398), CRF 67_01B (3.02%, 12/398) and CRF55_01B (2.51%, 10/398). The overall prevalence of drug-resistant mutations in cases with ART failure was 64.57% (257/398), including 45.48% (181/398) for nucleotide reverse transcriptase inhibitors (NRTIs) mutations, 63.32% (252/398) for non-nucleoside reverse transcriptase inhibitors (NNRTIs) mutations, and 3.02% (12/398) for protease inhibitors (PIs) mutations. Ten near full-length genomes (NFLG) of HIV-1 viruses were identified, including six recombinants of CRF 01_AE and subtype B, two recombinants of CRF 01_AE, subtype B and subtype C sequences, one recombinant of CRF 01_AE and subtype C and one recombinant of CRF 01_AE, subtype A1 and subtype C.

**Conclusions:**

The high prevalence of drug-resistant HIV-1 viruses was a serious challenge for HIV prevention and treatment of people with HIV infection. Treatment regimens for ART failure patients should be adjusted over time based on the outcome of drug resistance tests. NFLG sequencing facilitates the identification of new recombinants of HIV-1.

## Introduction

Human immunodeficiency virus (HIV) is a type of retrovirus that attacks T-helper cells of the human immune system and can cause acquired immunodeficiency syndrome (AIDS) [1]. Since HIV first emerged in the United States in 1981, the virus has spread worldwide and has become one of the most terrible health threats to humanity [2]. HIV-1 is the most common type circulating worldwide, while HIV-2 is prevalent in West Africa [3, 4]. HIV-1 strains can be further divided into four subgroups: M, O, N, and P [5]. The M group, accounted for the vast majority of the global epidemic, can be further classified into multiple subtypes (A, B, C, D, F, G, H, J and K) and many Circulation Recombinant Forms (CRFs) [6–8]. As we all know, highly active antiretroviral therapy (HAART) can help to reduce the viral load of HIV, maintain the immunity of patients, depress the incidence and mortality of AIDS [9, 10]. However, the risk of HIV drug resistance also increases with long-term use of antiviral therapy. The emergence and spread of drug-resistant mutants have not only result in failure of treatment, but also cause waste of public medical resources and seriously endangering public health safety, especially in developing countries [11]. Surveillance of drug resistance mutations can serve as a reference for the formulation of highly active antiretroviral therapy (HAART) treatment programmes.

Suzhou City suffers greatly from HIV infection. Since the first case of HIV infection was identified in Suzhou City in 1992, the number of confirmed HIV/AIDS cases has increased annually. Former HIV-infected people have progressively entered the onset phase of AIDS, and the number of AIDS-related deaths is on the rise. With the proliferation of drug-resistant strains, the situation of prevention and control of HIV infections is becoming increasingly serious in Suzhou City. However research on drug-resistant mutations in the HIV subtypes/CRFs is rare in Suzhou City. To clarify the questions, we identified and analyzed the *pol* gene sequences of HIV viruses from patients receiving ART for more than 1 year. Besides, we sequenced and characterized 10 near full-length genomes of HIV-1 recombinants.

## Material and methods

### RNA extraction and Sanger sequencing

Viral RNA was extracted from 200 µl plasma sample following the instructions of the MagNA Pure Compact Nucleic Acid Isolation Kit I (Roche, Switzerland). The partial *pol* gene fragment of HIV-1 virus was amplified by using in-house polymerase chain reaction (PCR) method as described previously [12, 13]. SuperScript™ III One-Step RT-PCR System with Platinum™ Taq DNA Polymerase (Invitrogen, USA) was used for reverse transcription reaction and first round PCR. The second round of PCR amplification was performed using Premix Ex Taq™ Version 2.0 (Takara, Japan) with a total volume of 50 µl. The length of the amplified product was 1.3 kb (HXB2:2147 to 3462), including the full length of the protease (PR) gene (1–99 codon) and the first 300 amino acids (1–300 codon) of the reverse transcriptase (RT) gene. The sequences of *pol* gene fragments were obtained by Sanger sequencing method.

### Genomes amplified and NGS

The near full-length genomes of recombinant forms of HIV-1 were amplified using the methods described previously [14, 15]. We mixed all amplified products of the same sample in one tube. The combined PCR products were purified by using AMPure XP purification beads. Nextera DNA Sample Prep Kit (Illumina, USA) was used for sequencing libraries prepared. Next generation sequencing (NGS) was performed on iseq platform by using i1 V2 300 cycles reagent kit (Illumina, USA).

### Sequence analysis

The Los Alamos National Laboratory HIV Sequence Database (http://www.hiv.lanl.gov/content/index) and REGA HIV subtyping tool (version 3.46, https://www.genomedetective.com/app/typingtool/hiv) were used for determining genotypes of HIV-1 viruses. The suspected new recombinant strains were submitted to Jumping Profile Hidden Markov Model (jpHMM-HIV) software (http://jphmm.gobics.de/submission_hiv). The Stanford HIV Drug Resistance Database system (https://hivdb.stanford.edu/hivdb/by-mutations/) was used to analyze resistance-related mutations and resistance levels by comparing with the sequence of wild type and drug resistant strains. Neighbor-joining (N-J) tree of the near full-length genomes of HIV-1 viruses was constructed by using MEGA 7.0 software with 1000 bootstrap replicates.

## Results

### Characteristic of subjects

From 2017 to 2020, 450 people living with HIV who have received antiviral therapy for more than 1 year were confirmed to be ineffective (viral load ≥ 1000 copies/ml) in Suzhou city. Among these ART failure patients, a total of 398 viral pol gene sequences were successfully sequenced. Epidemiological features of cases with ART failure in this study were summarized in Table 1. There was no significant difference in sex composition ratio, subtype distribution or viral load between group 2017–2018 and group 2019–2020. Males and females accounted for 92.96% (370/398) and 7.04% (28/398), respectively. A variety of HIV-1 virus circulating recombinant forms (CRFs) were prevalent in Suzhou City. The dominant subtype was CRF01_AE (57.29%, 228/398), followed by CRF07_BC (17.34%, 69/398), subtype B (7.54%, 30/398) and CRF08_BC (6.53%, 26/398). Other subtypes mainly included subtype CRF67_01B (3.02%, 12/398), CRF55_01B (2.51%, 10/398) and other CRFs (6.03%, 24/398). Among those who failed in antiviral treatment, the proportion of HIV-1 viral load in the range of 5000–100,000 copies/ml was the largest, accounting for 62.56% (249/398).

**Table 1:** The features of subjects receiving drug-resistance mutation detection in Suzhou City

Item	Total	2017–2018	2019–2020	χ^2^	*p*
		N (%)	N (%)		
Gender				1.80	0.17
Male	370	194	176		
Female	28	11	17		
Age (years)				2.086	0.35
< 30	107	61	46		
30–50	214	108	106		
≥ 50	77	36	41		
Subtypes/CRFs				2.34	0.89
CRF01_AE	228	118	110		
CRF07_BC	69	38	31		
Subtype B	30	13	17		
CRF08_BC	26	11	15		
CRF67_01B	12	7	5		
CRF55_01B	10	5	5		
Others	24	13	11		
Viral load (copies/ml)				3.84	0.15
1000–5000	110	48	62		
5000–100,000	249	135	114		
> 100,000	39	22	17		

### Drug-resistance mutations

**Fig. 1:**
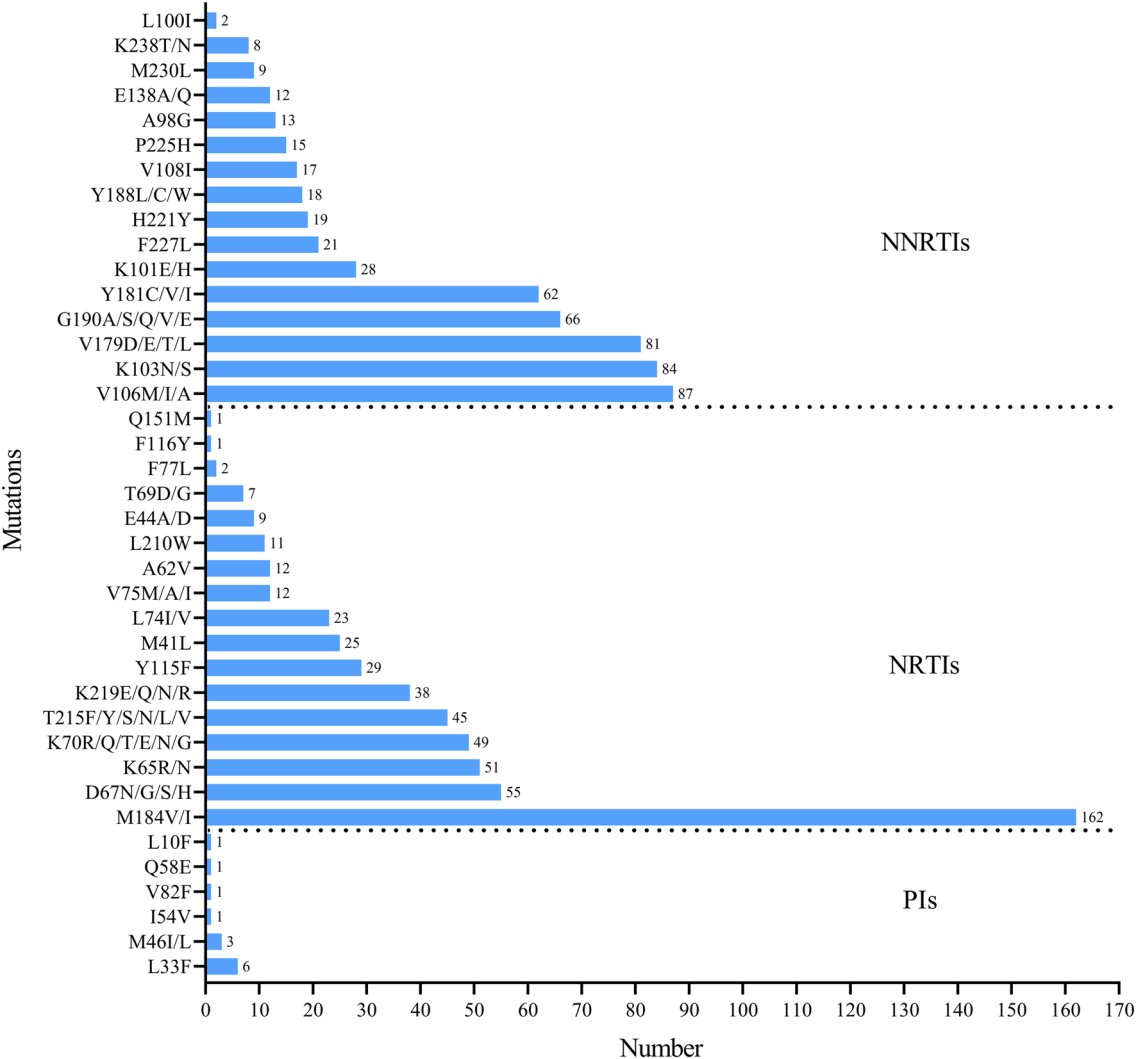
Frequency of drug-resistance mutations among cases with ART failure

According to the analysis of HIVdb Program, 257 strains were resistant to at least one drug of NRTIs, NNRTIs, or PIs, with an overall resistant rate of 64.57% (257/398). Frequency of drug-resistance associated mutations among cases with ART failure was shown in Fig. 1. The drug resistant mutations associated with NRTIs, NNRTIs, and PIs were 45.48% (181/398), 63.32% (252/398) and 3.02% (12/398), respectively. Among the PIs resistant HIV strains, four strains possessed PI-related major mutations (M46I/L, I54V, V82F) and the other 8 viruses had 3 accessory mutations (L33, FL10F and Q58E) in PR region. Respectively, 17 and 16 loci in RT region were found emerging NRTIs and NNRTIs resistance-associated substitutes. M184V/I (40.70%, 162/398), D67N/G /S/H (13.82%, 55/398) and K65R/N (12.81%, 51/398) were the three most common resistance-related mutations regarding to NRTIs, while V106M/I/A (21.86%, 87/398), K103N/S (21.11%, 84/398), V179D/E/T/L (20.35%, 81/398), G190A/S/Q/V/E (16.58%, 66/398) and Y181C/V/I (15.58%, 62/398) were the first five mutations associated with NNRTIs-resistance.

### Resistance level to antiviral drugs

**Fig. 2:**
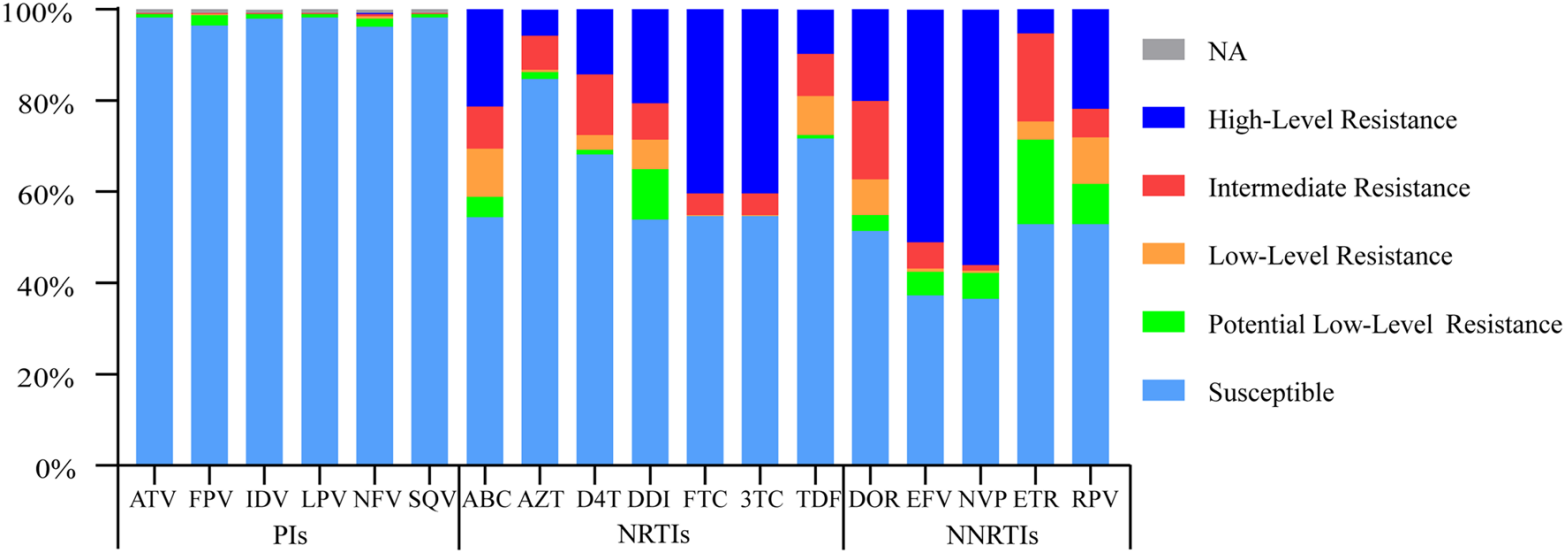
Resistance level of different drugs among cases with ART failure

We analyzed the effects of mutations on drug resistance based on Stanford University HIV drug resistance database (https://hivdb.stanford.edu/). No other HIV strains with high-level resistance to PIs were detected except 2020-SZ-83050 strain. Three samples (2017-SZ-08303, 2018-SZ-08066 and 2020-XC-00107) with M46I/L mutation were resistant to ATV, FPV, IDV, LPV, NFV and SQV at a potential low level or intermediate. Besides, six samples were simultaneously resistant to FPV, NFV and TPV at a potential low level caused by L33F mutation, and one sample with L10LF mutation was resistant to NFV and FPV at low level. Resistance levels of different drugs among ART failure individuals were shown in Fig. 2. For NRTIs, the resistant frequency to ABC, AZT, D4T, DDI, FTC, 3TC and TDF were 45.48% (181/398), 15.08% (60/398), 31.66% (126/398), 45.98% (183/398), 45.23% (180/398), 45.23% (180/398) and 28.14% (112/398), respectively. The high-level of NNRTIs associated resistance was accounting for 20.10% (80/398), 51.26% (204/398), 56.28% (224/398), 5.28% (21/398), and 21.86% (87/398) for DOR, EFV, NVP, ETR, and RPV, respectively.

### Analyses of NFLG sequences

**Fig. 3:**
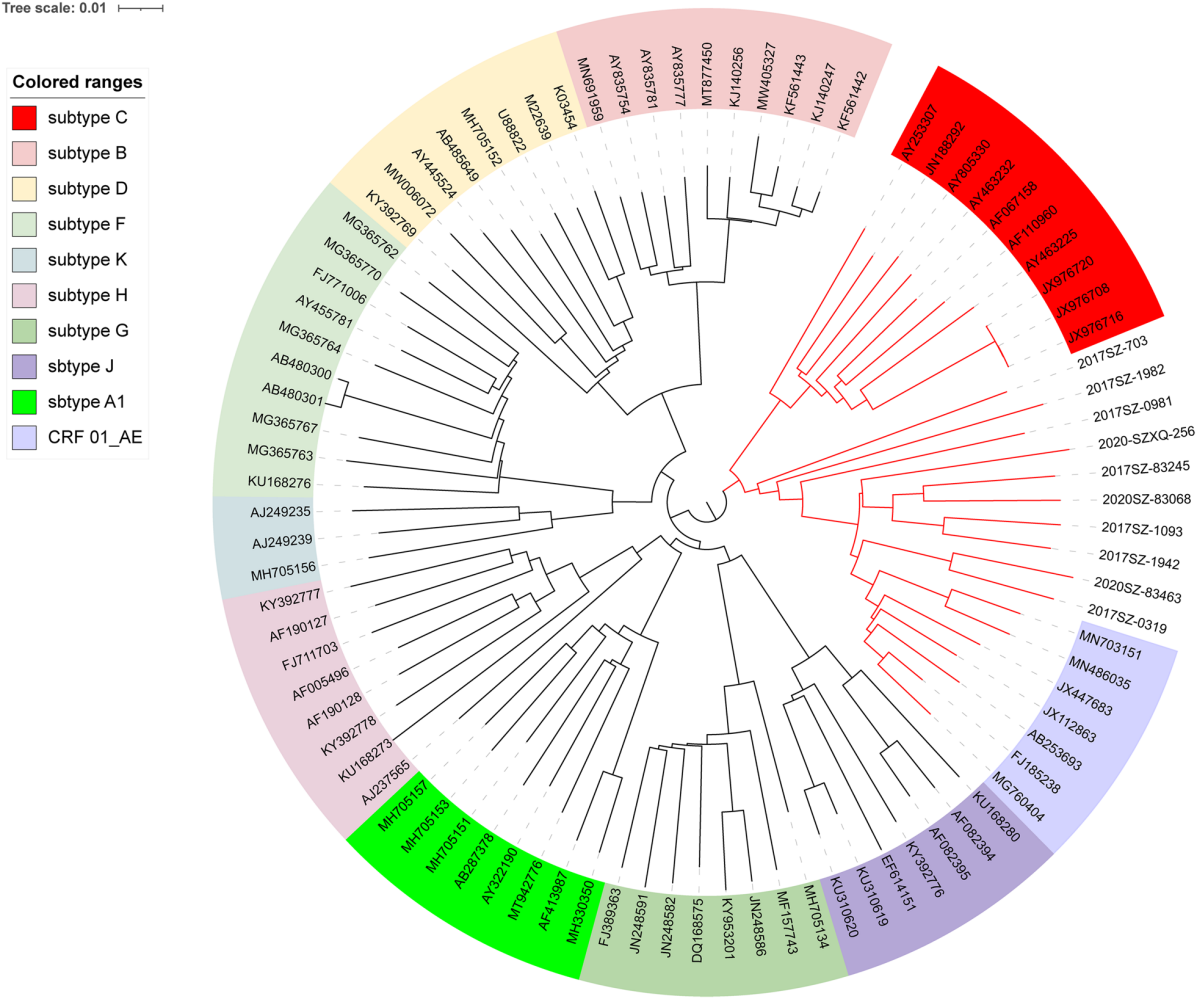
The Neighbor-joining phylogenetic tree of 10 near full-length genomes of Suzhou HIV-1 recombinant viruses. Uncolored labels represent Suzhou HIV-1 viruses

**Fig. 4:**
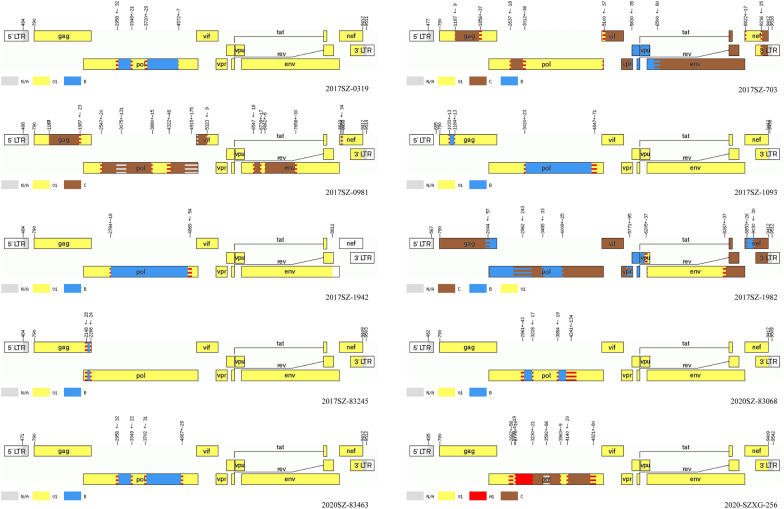
Recombinant analysis of 10 near full-length genomes of HIV-1 viruses in Suzhou City, China

Ten near full-length HIV-1 genomes (NFLG) covering from gag to nef genes were obtained by using next-generation sequencing (NGS). The Neighbor-joining phylogenetic tree of NFLG of HIV-1 viruses was shown in Fig. 3. According to the results of jpHMM-HIV software (Fig. 4), six sequences (2017SZ-83245, 2017SZ-0319, 2017SZ-1093, 2017SZ-1942, 2020SZ-83068 and 2020SZ-83463) were classified as recombinants of CRF 01_AE and subtype B. They were also supported by phylogenetic analysis (Fig. 3), because these sequences were closer to CRF 01_AE than subtype B. They were inter-subtype of small fragments of subtype B inserted in CRF 01_AE gene sequence, but the recombination breakpoints of every sample were different (Fig. 4). 2017SZ-703 and 2017SZ-1982 were identified as recombinants of CRF 01_AE, subtype B and subtype C. Sequences 2017SZ-703 and 2017SZ-1982 were closer to C subtype than others, indicating that they were recombinant viruses with subtype C virus as the backbone while CRF 01_AE and subtype B gene fragments as internal insertions. 2017SZ-0981 and 2020-SZXQ-256 sequences were recombinants of CRF 01_AE and subtype C.

## Discussion

With the increasing number of patients receiving antiviral treatment and the lengthening of antiviral treatment time, the risk of drug-resistant mutations will also increase and eventually lead to treatment failure. The viral load in people receiving treatment is high, suggesting that there is a potential for drug resistance [16]. Besides, HIV viruses, which were sensitive to ART, could be converted to drug-resistant strains under the influence of body immunity, drug selection pressure and other factors, such as poor compliance and incorrect drug dose [17, 18]. Studies have shown that some resistance-related substitutions will make the HIV-1 virus resistant to multiple drugs and increase the risk of spread to others [19]. HIV-1 viruses did not possess the drug resistant-related amino acids indicating that these strains probably exhibit sensitivity to corresponding drugs. However, among all the identified strains, 3.02%, 45.48% and 63.32% HIV-1 viruses were confirmed resistance to PIs, NRTIs and NNRTIs. The drug resistance rates of NRTIs and NNRTIs were similar to previous studies in other countries [18, 20]. For example, the resistance rates of NRTIs and NNRTIs were 83.0% and 88.7% among treatment failures in Philippines. The regimen consisted of TDF/AZT, 3TC and EFV/NVP is currently the most common acquired free first-line treatment in China. Unfortunately, 28.14%/15.08%, 45.23%, 51.26%/56.28% of HIV-1 virus strains have a certain degree of resistance to the above drugs, respectively. The high prevalence of drug-resistant HIV may lead to treatment failure and further spread, which will be detrimental to epidemic control. It is necessary to carry out drug resistance testing in time and adjust the treatment plan according to the results.

The distribution of HIV subtypes varies considerably across regions [5]. In this study, at least 8 subtypes/CRFs were identified. The subtypes/CRFs distribution of HIV-1 in Suzhou City is similar to that in other areas according to previous studies [21–24], with CRF01_AE accounting for the majority, followed by CRF07_BC, subtype B and other subtypes. However, some other surveys in China showed different results, such as that in Yunnan province [25] and Minority Area [26]. Besides, it was also different from that in Sierra Leone, Africa [27] or Florida, American [28], with CRF 02_AG and subtype B as the most common genotype, respectively. The HIV virus may benefit from the M46I, L33F and L10F substitutions, which can improve its resistance to PIs. In this study, V106M/I/A, K103N and V179D/E/T/L were the first three common mutations associated with NNRTIs resistance while M184V/I, D67N/G/S/H and K65R/N were the first three common mutations associated with NRTIs. These mutations were also high-frequency reported previous literatures [18, 29–31].

The probable emergence and spread of novel resistant strains deserve great attention. If viral genomes were recombinant and diverse, they could not be completely characterize using partial genomic sequences. Full genome sequencing can be used to confirm recombinants when viruses typing were unclassified or the results of different gene segments were inconsistent. A number of new HIV recombinants have been reported by near-full-length genome sequencing [32–35]. In this study, 12 recombinants were confirmed by performing full genome sequencing. All recombinant strains contained CRF 01_AE gene fragments. This may be related to the high prevalence of CRF 01_AE in the local area.

In conclusion, because of the severity and high fatality rate of HIV infections, the prevalence of drug resistant mutations HIV has aroused widespread social concern and posed a threat to public health. It is necessary to continuously monitor CD4^+^ T cell counts, viral load and drug-resistance mutations in order to timely adjust the medication regimen during antiviral therapy. The characteristics of genotypes and drug-resistance of the HIV-1 virus in Suzhou City were preliminary described in this study, but more and in-depth researches are required to provide more accurate scientific basis for HIV-infected prevention and control.

## Data Availability

The epidemiological data and *pol* gene sequences of this article are available in the drug resistance database of the National Center for AIDS/STD Disease Control and Prevention, China CDC.
